# An Optoelectronic thermometer based on microscale infrared-to-visible conversion devices

**DOI:** 10.1038/s41377-022-00825-5

**Published:** 2022-05-07

**Authors:** He Ding, Guoqing Lv, Xue Cai, Junyu Chen, Ziyi Cheng, Yanxiu Peng, Guo Tang, Zhao Shi, Yang Xie, Xin Fu, Lan Yin, Jian Yang, Yongtian Wang, Xing Sheng

**Affiliations:** 1 Beijing Engineering Research Center of Mixed Reality and Advanced Display, School of Optics and Photonics, Beijing Institute of Technology, Beijing, 100081 China; 2 Department of Electronic Engineering, Beijing National Research Center for Information Science and Technology, Institute for Precision Medicine, Center for Flexible Electronics Technology, and IDG/McGovern Institute for Brain Research, Tsinghua University, Beijing, 100084 China; 3 School of Materials Science and Engineering, Tsinghua University, Beijing, 100084 China

**Keywords:** Inorganic LEDs, Imaging and sensing

## Abstract

Thermometric detectors are crucial in evaluating the condition of target objects spanning from environments to the human body. Optical-based thermal sensing tools have received extensive attention, in which the photon upconversion process with low autofluorescence and high tissue penetration depth is considered as a competent method for temperature monitoring, particularly in biomedical fields. Here, we present an optoelectronic thermometer via infrared-to-visible upconversion, accomplished by integrated light receiving and emission devices. Fully fabricated thin-film, microscale devices present temperature-dependent light emission with an intensity change of 1.5% °C^−1^ and a spectral shift of 0.18 nm °C^−1^. The sensing mechanism is systematically characterized and ascribed to temperature dependent optoelectronic properties of the semiconductor band structure and the circuit operation condition. Patterned device arrays showcase the capability for spatially resolved temperature mapping. Finally, in vitro and in vivo experiments implemented with integrated fiber-optic sensors demonstrate real-time thermal detection of dynamic human activity and in the deep brain of animals, respectively.

## Abstract (web summary)

A thermosensitive optoelectronic upconversion device based on integrated microscale LED and photodiode structures, with temperature dependent of spectral shift and intensity change.

## Graphical abstract

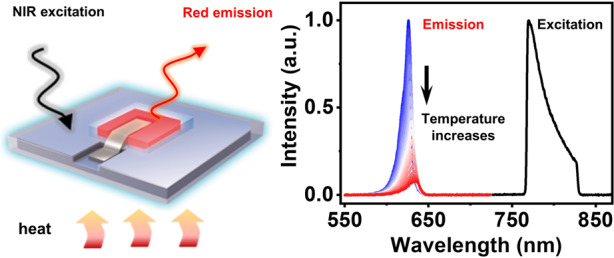

## Introduction

Spatially and temporally resolved temperature sensing with high precision is critically important and has wide applications in diverse fields, such as industrial manufacturing, environmental, and healthcare monitoring^1–5^. Specifically, real-time detection of temperature variations in biological systems is essential for point-of-care diagnostics and treatment^5–7^. Point contact temperature monitoring is commonly based on thermoelectric or thermoresistive sensors, but such wired electrical designs usually incorporate metallic embodiments that are easily intervened by electromagnetic waves, particularly during magnetic resonance imaging (MRI)^1,8^. On the other hand, optical-based sensors offer attractive solutions in temperature monitoring for biomedical diagnosis, owing to their remote detection, minimal invasion, immunity to electromagnetic interferences, and high resolution^1,9–13^. These optical sensing modalities can be based on the luminescence intensity, wavelength, peak width, and/or decay lifetime^1,11,12^. Infrared thermometers and imagers capture spatially resolved temperature information in a non-contact mode by collecting blackbody radiation emitted from the targeting object, but the devices only detect surface temperatures, and the results are highly influenced by surface emissivity^13,14^. Thermal sensors based on optical cavities exhibit spectrally resolved optical responses with very high precision^3,8^, but their measurements usually rely on sophisticated spectrometric systems in stable fixtures that could limit their biomedical applications. Phase changing materials like liquid crystals also present temperature-dependent color changes, but their perceived color can be limited by the environment lighting, viewing angles, polarizations, etc^15–17^.

Alternatively, temperature readout can be aided by the deployment of photoluminescent (PL) materials or devices, with the thermal status influencing their emission intensity, peak wavelength, decay lifetime, etc.^9–12,18–20^. These thermally dependent luminescent sensors provide visualized temperature information with a high spatiotemporal resolution, offering tremendous advantages and opportunities in areas like bioimaging^9–12,20,21^. While the temperature-dependent PL mechanism via the downconversion process requires a short-wavelength excitation, upconversion processes that transform near-infrared (NIR) photons in the range of the biological transparency window (around 650–950 nm) to visible ones are more advantageous and have emerged as an area of interest, particularly for biomedical applications^9,11,22–27^. Compared to the downconversion counterpart, such an upconversion mechanism mitigates the biological autofluorescence, facilitates tissue penetration, and yields conveniently-visualized and easily-captured visible light signals, presenting a more suitable method for sensing in biological systems^9,11,23–26,28^. As a representative and the most commonly used upconversion material, lanthanide-based nanoparticles leverage the PL emissions from two or more independent bands that exhibit strong temperature dependence, which have been applied for thermal sensing in biomedicine^19,23,25,29^. Recently, optoelectronic NIR-to-visible upconversion devices based on designed semiconductor heterostructures have been developed, exhibiting a linear response, fast dynamics, and low excitation power^30–32^. As a microscale device that can be implanted into the animal body, such an optoelectronic design shows promise for various optical sensing applications.

In this study, we systematically investigated temperature-dependent PL characteristics of the optoelectronic upconversion device and demonstrated its capability for thermal sensing. We discovered that its thermal-dependent PL emission is determined by the band properties of semiconductor materials as well as the integrated device circuit architecture. Arrays of patterned devices present spatially resolved temperature mapping in ambient environments. Furthermore, microscale devices integrated with fiber optics are employed for in vitro and in vivo applications, dynamically monitoring human exhalation activities and temperature variations in the deep brain of behaving animals.

## Results

**Fig. 1:**
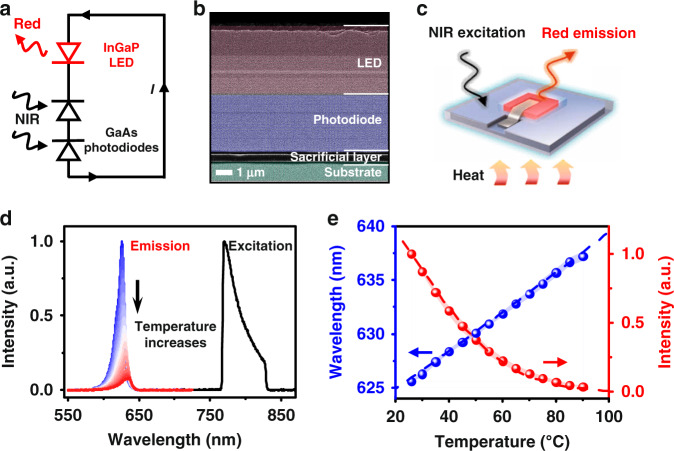
An optoelectronic upconversion device with temperature dependent light emission. **a** Circuit diagram and (**b**) Scanning electron microscopic (SEM) image of the optoelectronic upconversion design, including an InGaP red LED and a GaAs double junction photodiode with serial connection. **c** Schematic diagram of the upconversion device for temperature sensing. **d** Spectra of the excitation and the upconverted photoluminescence (PL) emission at varied temperatures (25–90 °C). **e** Calculated (dash line) and measured (dots) peak wavelength and PL intensity of the upconverted red emission as a function of temperature, and the shaded range represents the standard deviation measured among 10 samples

The proposed temperature sensing strategy is based on a fully integrated optoelectronic upconversion device schematically shown in Fig. 1a, consisting of a low-bandgap, gallium arsenide (GaAs) based double junction photodiode and a large-bandgap, indium gallium phosphide (InGaP) based light-emitting diode (LED) connected in series, with the band structure in Fig. S1. Figure 1b displays the cross-sectional scanning electron microscope (SEM) image of the device structure, which was grown on the GaAs substrate with a sacrificial interlayer. As demonstrated previously, the lithographically defined and epitaxially released microscale devices (size ~300 × 300 μm^2^) realize efficient NIR-to-visible upconversion with a linear response and ultrafast dynamics (Fig. 1c)^31,32^. In particular, here we find that the devices’ PL emission exhibit strong temperature dependence. Figure 1d depicts the spectroscopic performance of the device under steady-state NIR excitation at a wavelength range of 770–830 nm (power density ~40 mW cm^−2^, without causing additional photothermal effects in the tissue, as shown in Fig. S2)^33^, with red emission recorded by a fluorescence microscope equipped spectrometer (details in Fig. S3). Potentially used in the biomedical field, a temperature range from room temperature (~25 °C) to 90 °C is selected, in which the compositional materials (III–V semiconductors, encapsulants, and metal electrodes) in the device are stable. When the device temperature increases, the PL intensity decreases, accompanied by a redshift with the emission peak increasing from 625 nm to 637 nm. Device performance collected from 10 different devices is presented in Fig. 1e and S5. The measurements indicate an intensity-temperature sensitivity of ~1.5% °C^−1^ and a spectrum-temperature sensitivity of ~0.18 nm °C^−1^. Here the intensity-temperature sensitivity is subjected to the relative intensity, as the collected PL intensity is dependent on various factors, including the excitation power, efficiency of the spectrometer, device geometry, etc. On the other hand, the temperature-dependent spectral peak shift is subjected to the absolute sensitivity, in which the emission peak wavelength of the device depends on the bandgap of the InGaP semiconductor, regardless of other external factors. These experimental results are in quantitative accordance with theoretical calculations based on the detailed balance model for diodes and the empirical Varshni expression on the relationship between bandgap energy and temperature (see Supplementary Information for detailed calculations)^34–36^. We further analyze the noise associated with the spectral reading in Figure S6. Recorded at 27 °C, 48 °C, 65 °C, and 90 °C averaged signal-to-noise ratios (SNR) in the peak wavelength range (±5 nm) is above 15, larger than the 3 dB boundary. Taking the standard deviation of the normalized intensity at these representative temperatures, calculated temperature resolutions are 0.01 °C, 0.06 °C, 0.14 °C, and 0.49 °C, respectively. Similarly, resolutions of 0.04 °C, 0.08 °C, 0.11 °C, and 0.22 °C are achieved based on the peak wavelength shift at these representative temperatures.

**Fig. 2:**
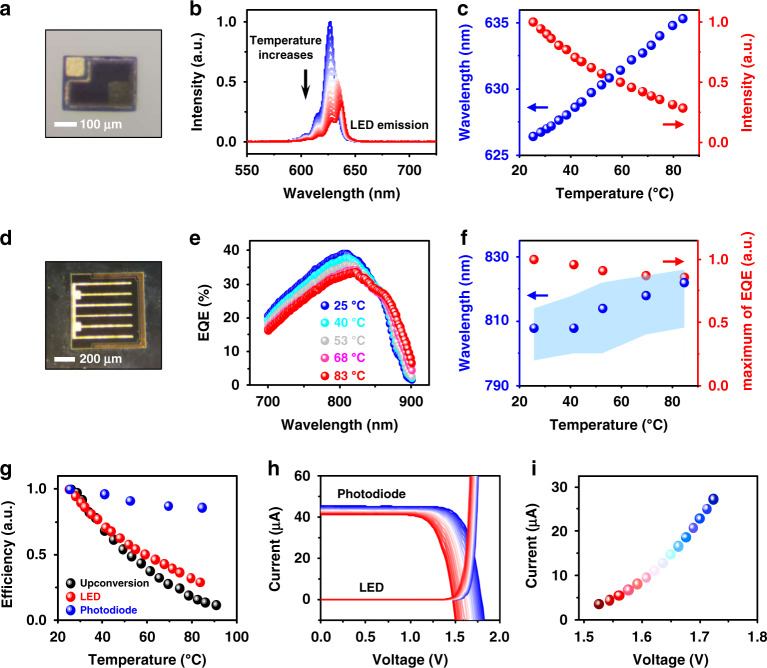
Thermal behaviors of individual InGaP LED and GaAs photodiode. **a** Micrograph of an InGaP LED. **b** Electroluminescence (EL) spectra of the InGaP LED at varied temperatures (25–90 °C), with an injected forward current of 20 µA. **c** EL intensity changes (red dots) and peak wavelength shifts (blue dots) for the InGaP LED at different temperatures. **d** Micrograph of a double junction GaAs photodiode. **e** External quantum efficiency (EQE) spectra of the GaAs photodiode at different temperatures. **f** Peak absorption intensity changes (red dot) and peak wavelength shifts (blue dot) of the GaAs photodiode at different temperatures. The shaded blue region is the wavelength range where the absorption intensity is above 98% of the peak. **g** Summary of efficiency results, including PL of the upconversion device, EL of the LED, and EQE of the photodiode at varied temperatures (25–90 °C). **h** Current–voltage curves of the InGaP LED and the GaAs photodiode (under irradiance 40 mW cm^−2^, 810 nm) at varied temperatures from 25 °C (blue) to 90 °C (red). **i** Values of current and voltage at the intersection points in **h**, indicating the corresponding working conditions of the optoelectronic upconversion device

To further grasp the thermal sensing mechanism of the upconversion device, we analyze the properties of individual electronic components with the device structure. We fabricate microscale InGaP LEDs (Fig. 2a) and GaAs photodiodes (Fig. 2d) and evaluate their thermal behaviors, separately. These individual devices have the same epitaxial structures as those in the upconversion device and are lithographically patterned to form similar microscale geometries^31,32,37^. The temperature-dependent electroluminescent (EL) spectra of the InGaP LED (Fig. 2b, c) are similar to those measured in the upconversion device (Fig. 1d, e). First, the EL peak shift coincides with that of the upconversion device, since the peak wavelength is mostly associated with the bandgap of the InGaP semiconductor (Fig. S4). Second, the LED emission intensity also decreases at elevated temperatures; however, the intensity-temperature relative sensitivity (~1.2% °C^−1^) is lower than that of the upconversion device, because the upconverted PL intensity is defined by both the LED and the photodiode. Compared with the LED powered by a stable external current source, the LED in the upconversion device is powered by the GaAs photodiode with temperature-dependent efficiencies, which further reduces the PL output at elevated temperatures. Figure 2e plots the external quantum efficiency (EQE) spectra of the double junction GaAs photodiode. When temperature increases, the optical absorption edge exhibits a redshift due to the narrowed bandgap of GaAs. Additionally, the EQE maximum decreases and the spectral peak slightly moves to longer wavelengths (Fig. 2f), which can be ascribed to the photocurrent mismatch within the double junction photodiode at elevated temperatures. Figure 2g summarizes the efficiency drops for all the devices (LED, photodiode, and the upconversion device). These results reveal that the intensity-temperature dependence of the upconversion device is determined by the efficiency drops of both the LED and the photodiode, while its spectrum-temperature sensitivity is mostly determined by the InGaP bandgap narrowing. Moreover, the operating conditions of the series-connected LED and photodiode within the upconversion device circuit eventually determine the overall current flowing through the LED and the emission intensity. Figure 2h plots the current–voltage characteristics of the LED and the photodiode at temperatures from 25 °C to 90 °C. Due to the bandgap narrowing of GaAs and InGaP (Fig. S2), both the photodiode open-circuit voltage and the LED turn-on voltage decrease upon heating. In such an upconversion device circuit (Fig. 1a), both the current and the voltage of the LED and the photodiode should match each other. Therefore, the intersection points of the current–voltage curves in Fig. 2h define the working conditions of both devices, which are plotted in Fig. 2i. It is noted that the intersection points of the current–voltage curves are also dependent on other factors, including the device structure as well as the excitation conditions. As shown in Fig. S7, temperature-dependent intensity sensitivities can be tuned by changing the excitation intensity.

**Fig. 3:**
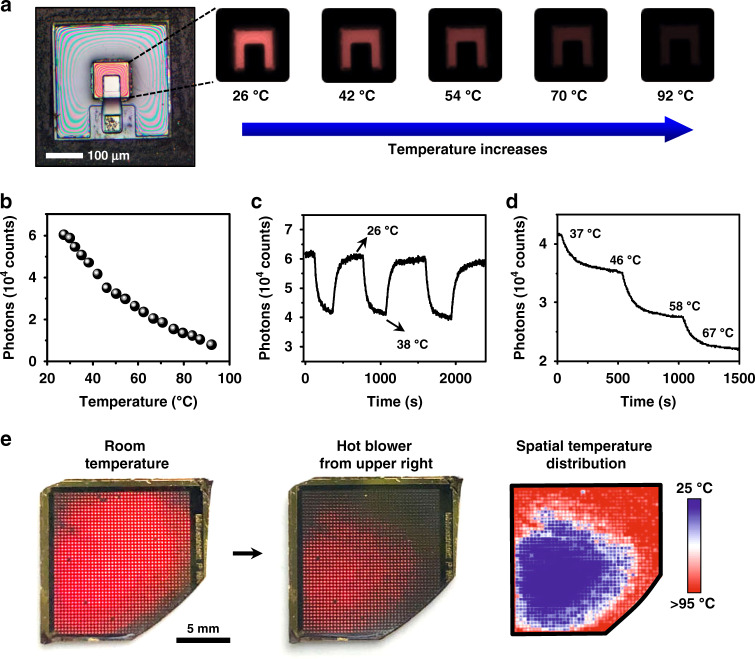
Dynamic thermal sensing and mapping based on the upconversion devices. **a** Microscopic images show PL emissions of an optoelectronic upconversion device, with the intensity changing with the temperature from 26 °C to 92 °C. **b** Relation of PL intensity versus temperature. **c** Cycled temperature test of the device repeatedly changes from 26 °C to 38 °C. **d** PL intensity of the device responding to step-tuned temperature changes. **e** Spatially resolved PL responses of a device array at room temperature (left) and under nonuniform heating (middle). Right: the corresponding temperature mapping

As previously demonstrated^31,32^, these NIR-to-red upconversion devices exhibit ultrafast PL dynamics with a decay lifetime of ~20 ns, enabling them to record temporally resolved thermal information. In addition, these devices can be lithographically patterned to form large arrays of various geometries with a high yield, which also makes spatially resolved temperature mapping feasible. Figure 3a illustrates a fully fabricated device, with PL mappings taken by a microscopic camera at various temperatures (mappings at more temperatures are shown in Fig. S8). Figure 3b plots the corresponding relation of PL intensity (measured by counting the averaged number of captured photons with an imaging sensor) and temperature, and the trend is similar to that obtained by a spectrometer (Fig. 1e). Figure 3c, d, and S9 demonstrate the dynamic response of the device during a cyclic heating/cooling process (between 26 °C and 38 °C) or a step rise at various temperatures. It should be noted that the long rise and decay time (>10 s) is mainly caused by the slow response of the electric heating plate, rather than the PL response of the device. In an environment with well controlled temperatures, the device presents a stable signal output, and PL signals are fully reversible under cyclic heating/cooling. Furthermore, we form an array of devices (size ~2 cm^2^, with ~1500 devices) to showcase the capability of spatially resolved thermal sensing (Fig. 3e and Movie S1). At room temperature, the device array presents a uniform red emission under NIR excitation. A hot airflow blows on the sample, disturbs and eventually extinguishes the emission when the surface temperature is above ~95 °C. The visualized thermal maps can be quantified based on the intensity-temperature calibration obtained in Fig. 3b. In terms of spatial resolution, the performance of the current array is determined by the device pitch (~300 μm), which can be further reduced to several micrometers by optimized lithographical processes. Since the technique records PL emissions at visible wavelengths (~625 nm), it potentially owns a spatial resolution much higher than conventional thermal imagers, which is based on mid-IR or far-IR absorbing materials and ultimately limited by the wavelength of blackbody radiation (10–20 μm at room temperature).

**Fig. 4:**
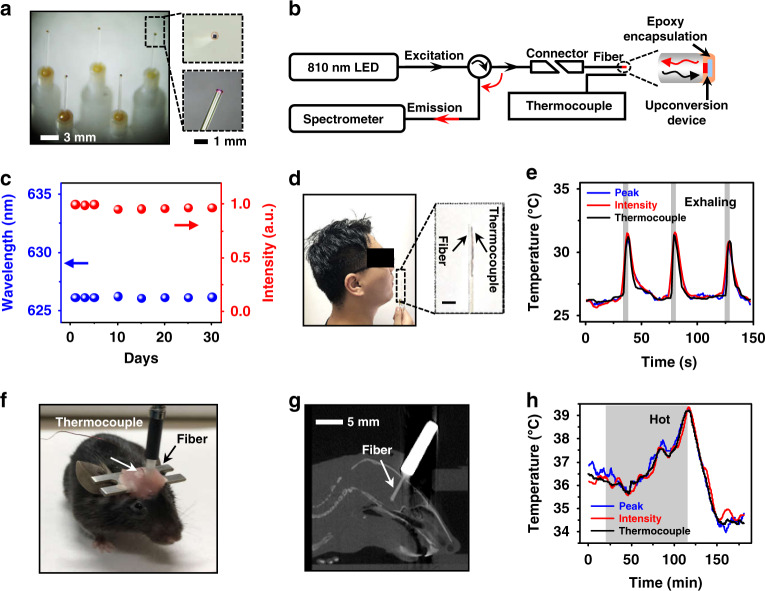
Optoelectronic upconversion devices integrated with fiber optics for in vivo temperature sensing. **a** Photographs of upconversion devices transferred on the tips of silica fibers (diameter ~600 µm). Enlarged boxes present a zoomed-in view of the fiber tip with a red-emitting device excited by the coupled near-infrared light. **b** Schematic illustration of the temperature sensing setup for spectral analysis, mainly including an 810 nm LED source, a spectrometer, and a Y-shaped fiber, as well as a thermocouple for temperature calibration. **c** Chronic PL stability of the encapsulated fiber sensor immersed in PBS for different times at room temperature (~25 °C), in which both the peak wavelength and PL intensity are recorded. **d** Photograph of the exhaling temperature detection with the fiber sensor. **e** Dynamic temperature signals during cycled exhaling activities obtained by the fiber sensor based on the emission peak wavelength shifts and PL intensity changes, compared with results simultaneously recorded by the thermocouple. The gray regions represent actions of exhaling. **f** Photograph of a behaving mouse with a fiber sensor and a thermocouple implanted into the brain for temperature sensing. **g** Sagittal CT reconstruction of a slice, indicating the location of the implanted fiber. **h** Dynamic temperature signals obtained in the mouse brain by the fiber sensor based on the emission peak wavelength shifts and PL intensity changes, compared with results simultaneously recorded by the thermocouple. The shaded gray region represents the time period when the mouse is placed in a hot environment at around 40 °C

The upconversion device can further be integrated with fiber optics to form light-guided thermal sensors. Epitaxial lift-off and transfer printing techniques produce thin-film, freestanding devices mounting on tips of quartz fibers (Fig. 4a, S10, and S11). Different from free space detectors, such integrated fiber sensors can be interfaced with standard optical setups and transmit all signals within the fibers. Figure 4b outlines the setup established for fiber-based temperature sensing, with a NIR excitation LED (~810 nm) and a spectrometer connected to the two branches of a Y-shaped fiber terminated by our upconversion fiber. In parallel, the optical system can employ a standard thermocouple for calibration and comparison. The encapsulated device on the fiber tip exhibit desirable stability when immersed in the phosphatebuffered saline solution (up to 30 days at room temperature), as well as ideal mechanical stability (Fig. S12). Such a fiber-coupled, portable system can be conveniently applied for biomedical applications, for example, monitoring the exhalation behavior closed to the mouth of a human, as a proof-of-concept demonstration (Fig. 4d). With the correlation between the PL intensity/peak wavelength and temperature (Fig. S13) established from the calibration with the thermal couple, the fiber sensor can monitor the change of exhaled temperature with time. As shown in Fig. 4e, S13, S14, and Movie S2, both curves match well with the results obtained by a colocalized thermocouple, showing determination coefficients *R*^2^ = 0.90.

The fiber-coupled device can also monitor the temperature fluctuations in the deep brain. While magnetic resonance based non-invasive measurements of the deep brain temperature are possible^38,39^, the accuracy and system complexity still limit their broad applications. Complementary with tethered electrical sensors, such an optical-based technique is more suitable for use in environments with strong electromagnetic interferences, and in particular, capable of obtaining signals during magnetic resonance imaging (MRI). As a consequence, some commercially available fiber optic temperature sensors^40,41^ based on the temperature-dependent optical properties, e.g., the bandgap of a GaAs crystal material, as the thermal transduction mechanisms have been put into practice^42^. Different from these semiconductor-based sensors via downconversion photoluminescence, our thin-film devices leverage the thermal behaviors of semiconductor bandgaps and diode junctions, which potentially provide improved sensitivities, and the upconversion mechanism minimizes tissue attenuation and autofluorescence associated with the use of visible excitation sources. Figure 4f shows a behaving mouse with a fiber sensor as well as a thermocouple inserted in the same brain region via standard stereotaxic surgery (details provided in methods). The reconstructed x-ray computed tomography (CT) images in Fig. 4g reveal that the fiber tip is located in the hypothalamus region. To minimize the thermal effect of the excitation source, a pulsed NIR light (810 nm, power 1 mW, frequency 0.1 Hz, pulse width 3 s) is applied to illuminate the upconversion device. We place the mouse in a temperature-controlled enclosure and dynamically record the optical response of the implanted fiber sensor while changing the ambient temperature. Similarly, temperature results can be extracted from both the PL intensity and peak wavelength variations. Figure 4h, S13, and S14 present the brain temperature simultaneously recorded by the fiber and the thermocouple in a period of 3 h, and the results indicate that these optical and electrical readouts are in good accordance with a determination coefficient *R*^2^ > 0.97. The brain temperature increases when the mouse is in a hot environment (~40 °C), and then drops to ~34 °C when the heat is turned off, due to the heat dysregulation response^43,44^. Therefore, this fiber-based thermal sensor can be exploited together with other neural modulation and interrogation methodologies like optogenetics and fluorescence photometry^7,45,46^, making it feasible to probe the local thermal condition of a specific brain region.

## Discussion

In summary, here we present an optical thermometer comprising integrated optoelectronic devices for photon upconversion, with an intensity-temperature sensitivity of 1.5% °C^−1^ and a large spectral shift of 0.18 nm °C^−1^, in the range of 20–90 °C. Compared to other upconversion materials like lanthanide nanoparticles, dyes, and quantum dots, our device provides an alternative approach for temperature sensing, featuring high sensitivities and low-power excitation. Based on similar semiconductor materials and architectures, large-area device arrays with a wafer-level scale and pitches of a few micrometers can be readily archived for thermal mapping with a high resolution. The MRI-compatible, implantable sensors combined with fiber optics offer both research and clinical significance, with a potential for localized temperature monitoring in the deep body. One limitation of the current device design is its red emission (~625 nm), which is not within the biological transparency window. Future design can involve semiconductors both absorption and emission at longer wavelengths. Overall, these materials and device concepts establish a power tool set with vast applications in the environment and healthcare.

## Materials and methods

### Device fabrication

Details about the structure and fabrication processes of the optoelectronic upconversion devices can be found in our previous work^31,32^. Via metal-organic chemical vapor deposition (MOCVD), the InGaP red LED and the double junction GaAs photodiode are grown on a GaAs substrate, with an Al_0.95_Ga_0.05_As sacrificial layer between the device layers and the GaAs substrate. The device geometry is lithographically patterned by selective wet etching and metallization. Thin-film, freestanding devices are formed by eliminating the sacrificial layer, and fully released devices are transferred printed onto the thermal release tape. The devices are detached from the heat release tape (3198 M, Semiconductor Equipment Corp.) by heating the tape to 120 °C. Pick up the detached device with the epoxy (SU8-3005) coated fiber tip, followed by encapsulation with ~20 μm thick polydimethylsiloxane (PDMS, Sylgard-184, Dow Corning, base: curing agent ratio = 10:1 w/w, cured at 80 °C for 2 h) and then ~10 μm thick parylene via chemical vapor deposition (CVD).

### Device characterization

Devices photographs are taken with an Olympus IX53 microscope equipped with a Xenon arc lamp, in which the excitation light and the emission light pass through a set of fluorescence filters (EX ET800/60, BS T700spxr-UF1, EM ET650sp, Chroma Tech. Corp.). The PL emission is collected by an Andor Zyla 4.2Plus CMOS camera (an area of ~20 μm^2^ is chosen, acquisition time 20 ms) or a spectrometer (HR2000+, Ocean Optics). Samples are placed on a ceramic electric heating plate for temperature control, which is calibrated by a thermocouple. The current–voltage characteristics of devices are measured with a Keithley 2400 source meter. The EQE spectra of photodiodes are collected by a standard system (QEX10, PV Measurement). A standard thermocouple (YET-620) with a T-type microprobe is used for temperature reference and system calibration.

### Animal studies

All animal procedures are approved by the Institutional Animal Care and Use Committee (IACUC) at Tsinghua University. Adult male C57BL/6 J mice (8–12 weeks) are purchased from the Vital River Laboratory Animal Technology (Beijing, China), and are used and housed under standard conditions in groups (3–5 mice per cage).

Following anesthesia with an intraperitoneal injection of 0.5% sodium pentobarbital (10 mL kg^−1^), the scalp is shaved and the mice are placed in a stereotaxic frame. A hole with a diameter of ~800 μm is drilled in the implantation site on the skull, the fiber sensor is slowly inserted into the hypothalamus (AP: 0.5 mm, ML: 0.5 mm, DV: −4.8 mm), and then the fiber sensor is secured to the skull by dental cement.

## Supplementary information

SUPPLEMENTAL MATERIAL

Movie S1

Movie S2
