# Supplementary material for: An Optoelectronic thermometer based on microscale infrared-to-visible conversion devices
Source: Light Sci Appl. 2022 May 7;11:130. doi: 10.1038/s41377-022-00825-5 (PMC9079085; doi:10.1038/s41377-022-00825-5)
Supplement: Supplementary file 1 — SUPPLEMENTAL MATERIAL [file 41377_2022_825_MOESM1_ESM.pdf]

Supplementary Information for

An Optoelectronic Thermometer based on Microscale Infrared-to-Visible Conversion Devices

He Ding[1, *], Guoqing Lv[1], Xue Cai[2], Junyu Chen[2], Ziyi Cheng[1], Yanxiu Peng[1], Guo Tang[2], Zhao Shi[2], Yang Xie[2], Xin Fu[3], Lan Yin[3], Jian Yang[1], Yongtian Wang[1], Xing Sheng[2,*]

[1]Beijing Engineering Research Center of Mixed Reality and Advanced Display, School of Optics and Photonics, Beijing Institute of Technology, Beijing, China

[2]Department of Electronic Engineering, Beijing National Research Center for Information Science and Technology, Institute for Precision Medicine, Center for Flexible Electronics Technology, and IDG/McGovern Institute for Brain Research, Tsinghua University, Beijing, China

[3]School of Materials Science and Engineering, Tsinghua University, Beijing, China

* Corresponding to: heding@bit.edu.cn; xingsheng@tsinghua.edu.cn

Calculations of the emission peak wavelength and intensity the upconversion device as a function of the temperature

The optical and electrical properties of semiconductor materials are highly susceptible to temperature, so the spectral response of optoelectronic upconversion devices can be determined by temperature changes (*1–3*).

As the temperature (T, in unit Kelvin) increases, energy band gaps (E_g) of the semiconductors (InGaP and GaAs) decreases, based on the empirical Varshni expression (*3*):

$$E_g(T) = E_g(0) - \frac{\alpha T^2}{T + \beta} \tag{1}$$

where $E_g(0)$ is the energy bandgap at $T = 0$ K, $E_{g\_InGaP}(0) = 2.07$ eV, $E_{g\_GaAs}(0) = 1.52$ eV, α and β are fitting parameters ($\alpha_{InGaP} = 4.8\times10^{-4}$ K^{-1}, $\beta_{InGaP} = 200$ K; $\alpha_{GaAs} = 5.4\times10^{-4}$ K^{-1}, $\beta_{GaAs} = 204$ K) from the literature (*3, 4*). Data are plotted in Figure S4 (blue curve).

The emission peak wavelength (λ) of the InGaP LED can be calculated as a function of the temperature:

$$\lambda(T) = \frac{hc}{E_g(T)} \tag{2}$$

where h is Planck's constant, c is the speed of light. Based on Eq.(2), we can calculate the wavelength vs. temperature plot in Figure 1e.

Based on the detailed balance theory (*2*), the output current from the GaAs photodiode I_{PD} is equal to the difference between generated carriers and recombined carriers:

$$I_{\mathrm{PD}} = I_{\mathrm{ph}} + I_{\mathrm{th}} - I_{\mathrm{rad}} - I_{\mathrm{nrad}} \tag{3}$$

where I_{ph} is the photogenerated current and derived from the excitation light (~40 mW cm^{-2}), I_{th} is the absorbed thermal radiation from the environment, I_{rad} is the radiative current, and I_{nrad} is the non-radiative current.

Similarly, the input current for the LED is mainly converted to the radiative recombination and non-radiative recombination process.

$$I_{\mathrm{LED}} = -I_{\mathrm{th}} + I_{\mathrm{rad}} + I_{\mathrm{nrad}} \tag{4}$$

For both the GaAs photodiode and the InGaP LED, the absorbed thermal radiative current is defined as:

$$I_{\mathrm{th}} = \frac{2\pi(n^2+1)qkTS}{h^3c^2} E_{\mathrm{g}}^2 \exp\left(-\frac{E_{\mathrm{g}}}{kT}\right) \tag{5}$$

where k is Boltzmann's constant, n is the refractive index, and S is the surface area of the device. In the temperature range of our interest (20–100 °C), the thermal currents are around 1×10^{-26} A m^{-2} (for InGaP) and 1×10^{-18} A m^{-2} (for GaAs), much smaller than the photocurrents (~1 A m^{-2}) under excitation. Therefore, the effect of thermal currents can be neglected in such an integrated device.

The radiative current is dependent on the voltage (V) and defined as:

$$I_{\mathrm{rad}} = I_{\mathrm{th}} \exp\left(\frac{qV}{kT}\right) \tag{6}$$

The radiative and absorption efficiencies of the LED and double junction GaAs photodiode are assumed to linearly drop from 100% to 30% and from 100% to 90%, respectively, from 25 °C to 100 °C, as shown in Figures 2c and 2f. The conversion efficiency (η) is highly dependent on the temperature changes, and the major fraction

of the carriers contribute to non-radiative recombination and carrier leakage, resulting in low efficiency (*5*). Taking these conversion efficiencies (η) of the diode from the experimental results, in which the I_{nard} is defined as:

$$I_{\text{nrad}} = \frac{I_{\text{rad}}}{\eta} - I_{\text{rad}} \tag{7}$$

Thus the I_{LED} and I_{PD} can be summarized as:

$$\begin{aligned} I_{\text{LED}} &= -I_{\text{th}} + I_{\text{rad}} + I_{\text{nrad}} \\ &= \frac{2\pi(n^2+1)qkTS}{h^3c^2}\left(E_{\text{g\_InGaP}}(0) - \frac{\alpha_{\text{InGaP}}T^2}{T+\beta_{\text{InGaP}}}\right)^2 \exp\left(-\frac{E_{\text{g\_InGaP}}(0) - \dfrac{\alpha_{\text{InGaP}}T^2}{T+\beta_{\text{InGaP}}}}{kT}\right)\left(-1 + \frac{\exp\left(\dfrac{qV}{kT}\right)}{\eta_{\text{LED}}}\right) \end{aligned} \tag{8}$$

$$\begin{aligned} I_{\text{PD}} &= I_{\text{ph}} + I_{\text{th}} - I_{\text{rad}} - I_{\text{nrad}} \\ &= I_{\text{ph}} + \frac{2\pi(n^2+1)qkTS}{h^3c^2}\left(E_{\text{g\_GaAs}}(0) - \frac{\alpha_{\text{GaAs}}T^2}{T+\beta_{\text{GaAs}}}\right)^2 \exp\left(-\frac{E_{\text{g\_GaAs}}(0) - \dfrac{\alpha_{\text{GaAs}}T^2}{T+\beta_{\text{GaAs}}}}{kT}\right)\left(1 - \frac{\exp\left(\dfrac{qV}{kT}\right)}{\eta_{\text{PD}}}\right) \end{aligned} \tag{9}$$

I_{LED} vs. V and I_{PD} vs. V under different illumination conditions are plotted in Figures 2h and S7a.

In the optoelectronic upconversion device, the double junction GaAs photodiode structure is connected in series to the InGaP LED structure. Under illumination, the current and voltage of the integrated design should satisfy:

$$\begin{cases} I_{\text{LED}} = I_{\text{PD}} \\ V_{\text{LED}} = V_{\text{PD}} - V_{\text{f}} - I_{\text{LED}}R_{\text{LED}} - I_{\text{PD}}R_{\text{PD}} \end{cases} \tag{10}$$

where V_{f} = 0.4 V is a fitting value representing the discrepancy between the theoretical and experimental output voltages of the double junction GaAs photodiode. R_{LED} = R_{PD} = 150 Ω are series resistances within the device circuit. By solving Eq. (10), we can obtain intensity vs. temperature (red curve) in Figure 1e.

Reference

1. Levinshtein, M., Rumyantsev, S. & Shur, M. Handbook Series on Semiconductor Parameters. (Singapore: World Scientific, 1996).

2. Shockley, W. & Queisser, H. J. Detailed balance limit of efficiency of *p-n* junction solar cells. *Journal of Applied Physics* **32**, 510-519 (1961).

3. Varshni, Y. P. Temperature dependence of the energy gap in semiconductors. *Physica* **34**, 149-154 (1967).

4. Vurgaftman, I., Meyer, J. R. & Ram-Mohan, L. R. Band parameters for III–V compound semiconductors and their alloys. *Journal of Applied Physics* **89**, 5815-5875 (2001).

5. Meyaard, D. S. *et al*. Temperature dependent efficiency droop in GaInN light-emitting diodes with different current densities. *Applied Physics Letters* **100**, 081106 (2012).

Figure S1

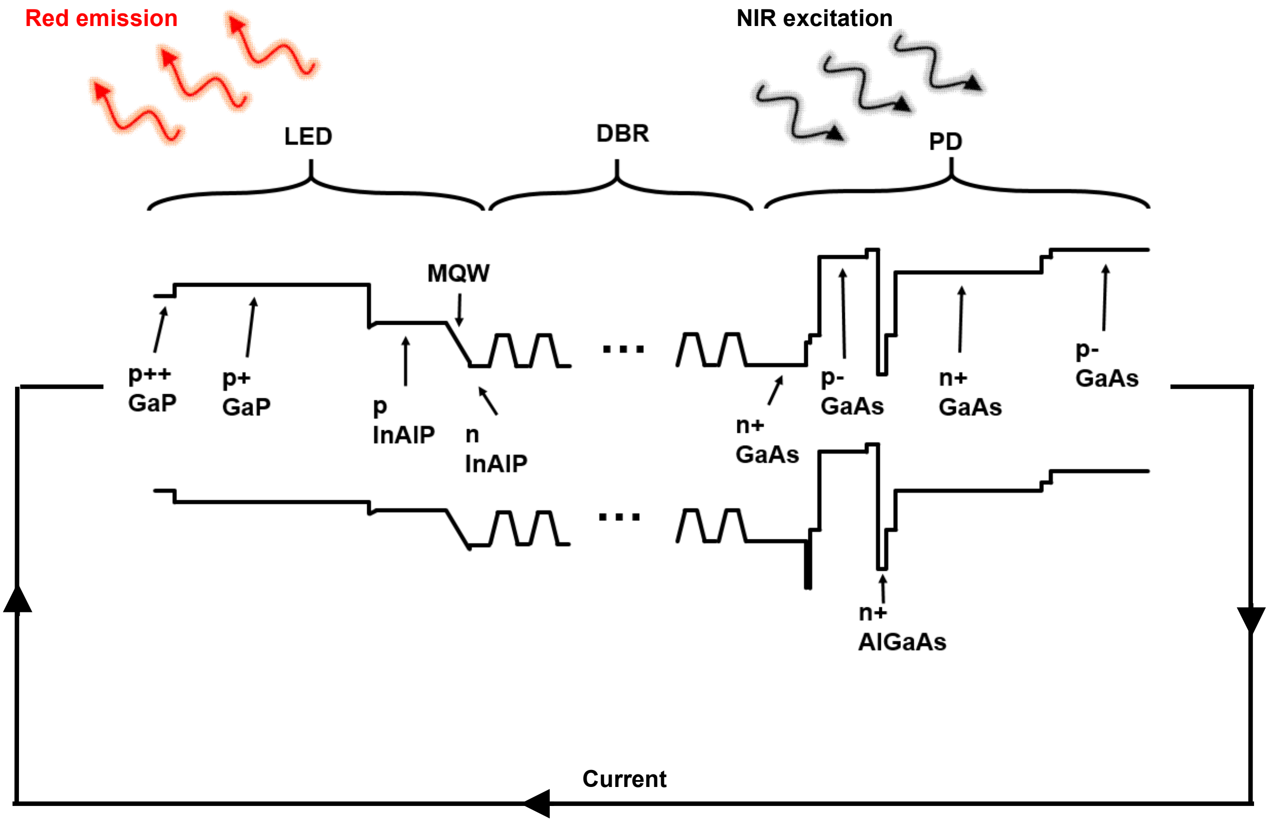

Figure S1. Schematic drawing of the band structure for our optoelectronic upconversion device.

Figure S2

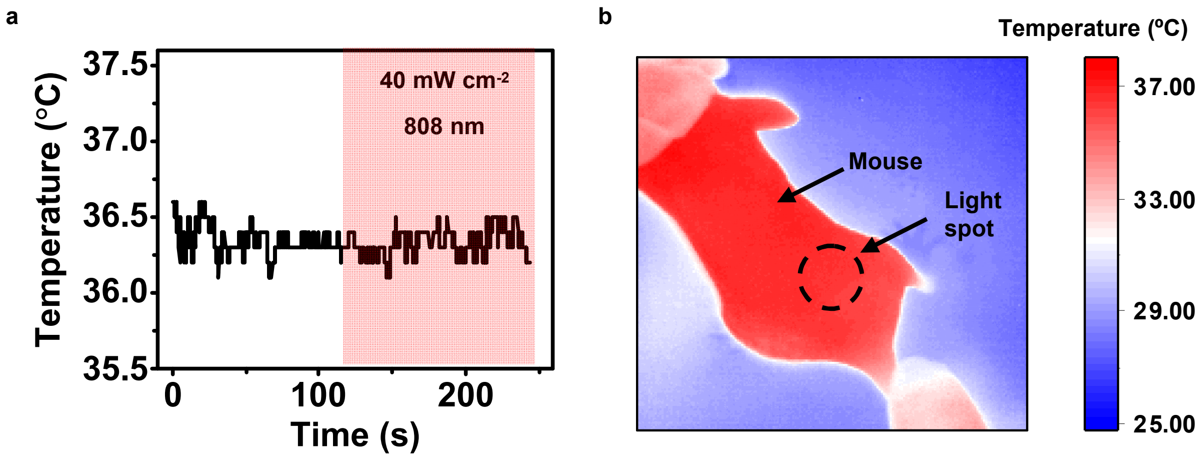

Figure S2. (a) Temperature response of the mouse skin under irradiation at ~40 mW cm^{-2} at 808 nm. (b) The dashed circle indicates the light spot on the mouse skin.

Figure S3

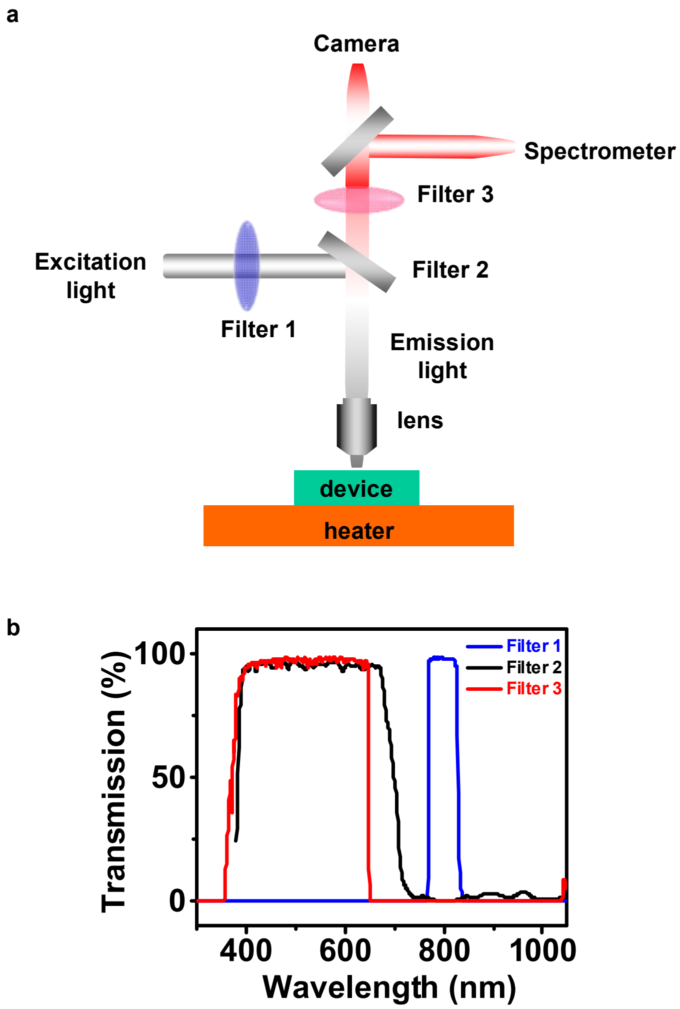

Figure S3. (a) Schematic diagram of the optical setup for temperature sensing based on PL changes of the optoelectronic upconversion device. (b) Optical transmission spectra of fluorescence filter combinations.

Figure S4

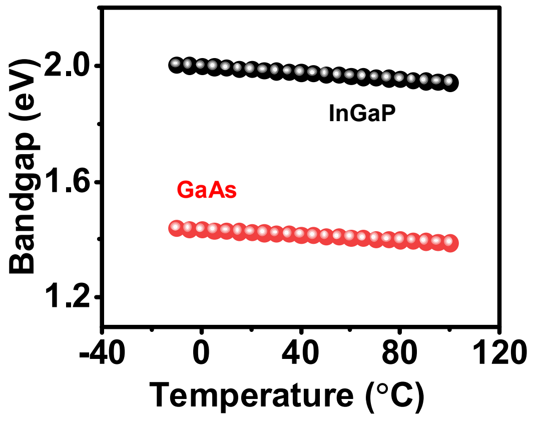

Figure S4. Bandgaps of InGaP and GaAs as a function of the temperature.

Figure S5

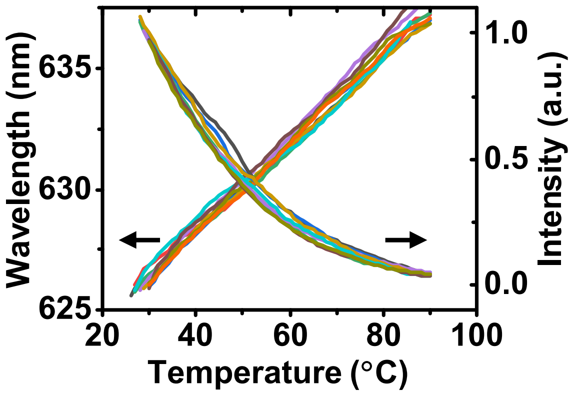

Figure S5. Measured peak wavelength and PL intensity of the upconverted red emission spectra as a function of temperature for a group of 10 different samples.

Figure S6

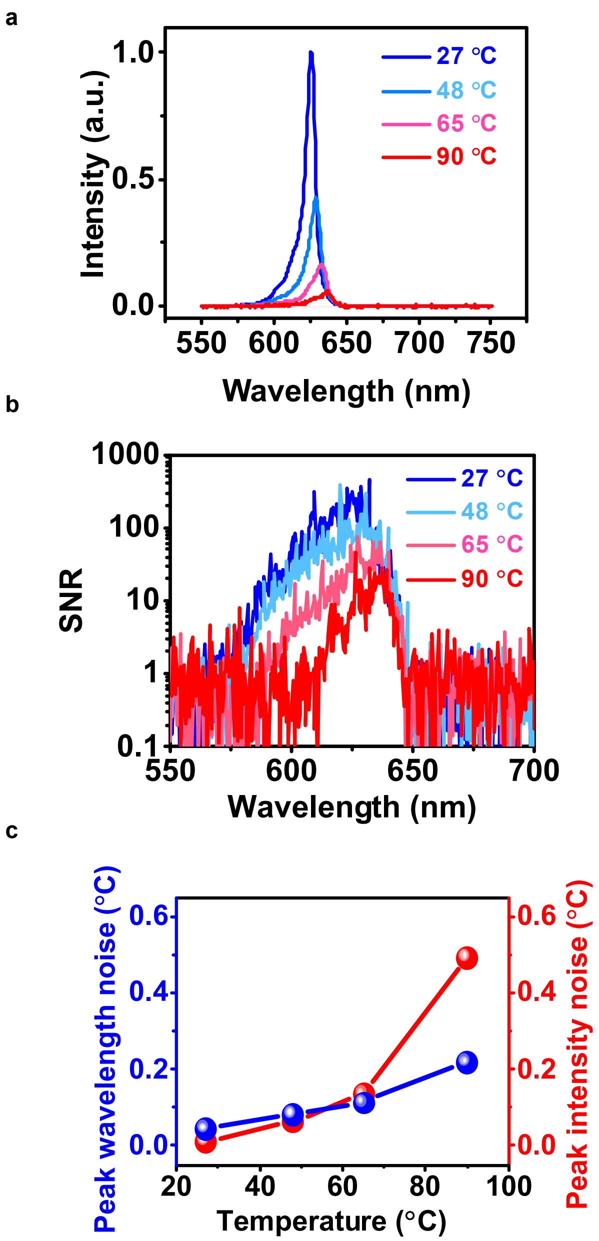

Figure S6. (a) Upconverted emission spectra and (b) signal to noise ratio (SNR) of the optoelectronic upconversion device at the representative temperatures of 27 ºC, 48 ºC, 65 ºC, and 90 ºC. (c) The temperature detection accuracy based on peak wavelength shift and intensity changes by noting the equivalency their standard deviation to temperature changes in Figure 2e.

Figure S7

a

b

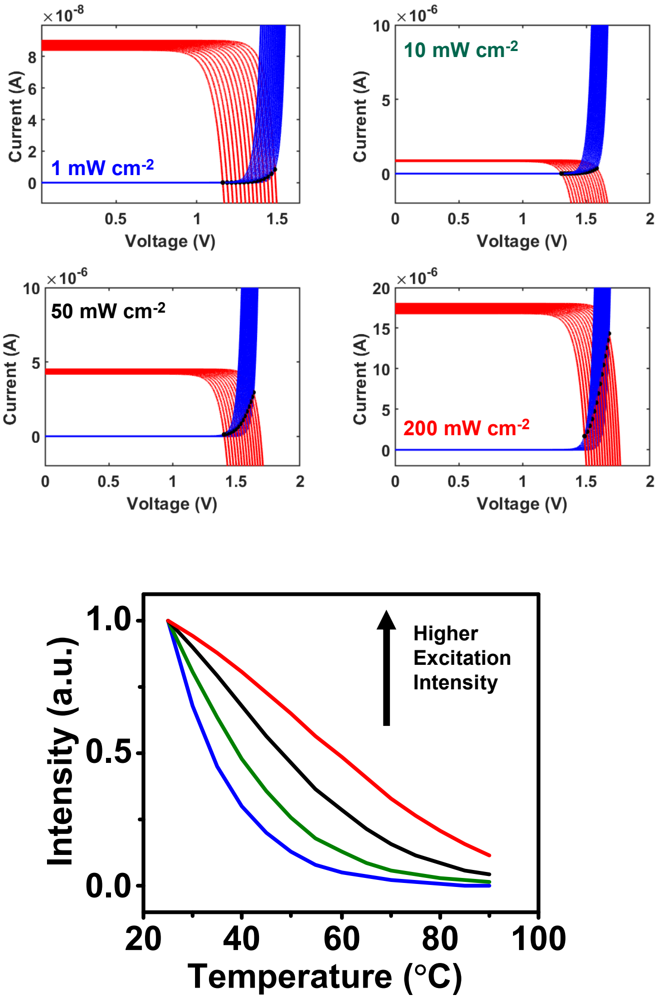

Figure S7. (a) Calculated current–voltage curves of the InGaP LED and the GaAs photodiode structures for the optoelectronic upconversion device at varied temperatures from 25 ºC to 90 ºC, under the different light intensities from 1 mW cm^{-2} to 200 mW cm^{-2}. (b) Corresponding temperature-dependent intensity changes.

Figure S8

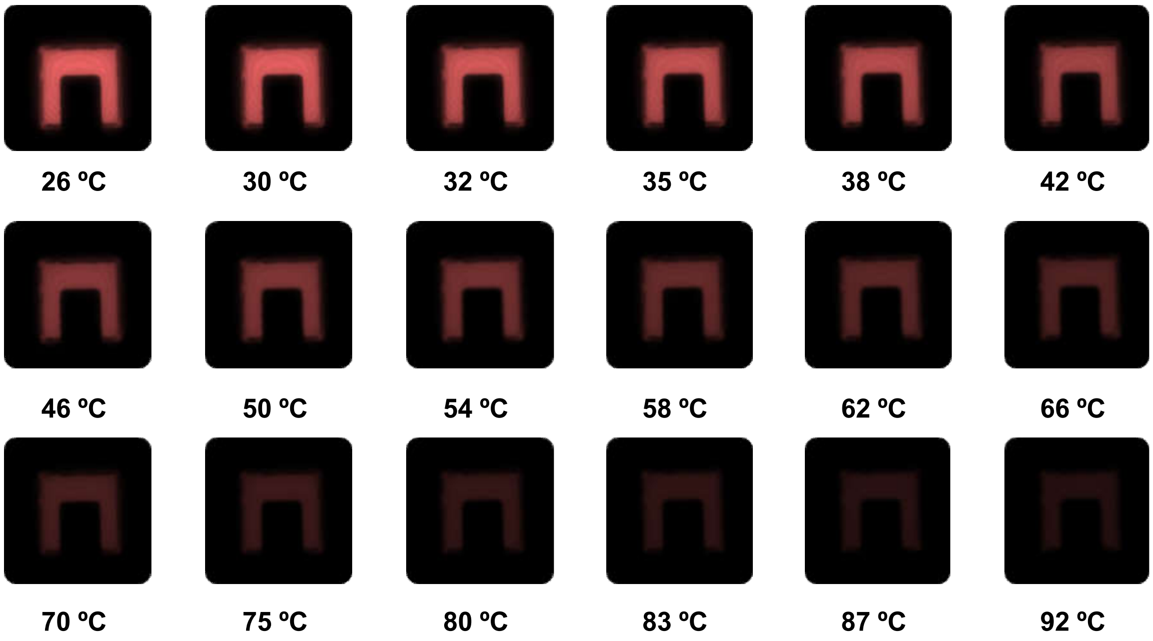

Figure S8. Photographs showing photoluminance intensity changes of an upconversion device, with the temperature increasing from ~26 ºC to 92 ºC.

Figure S9

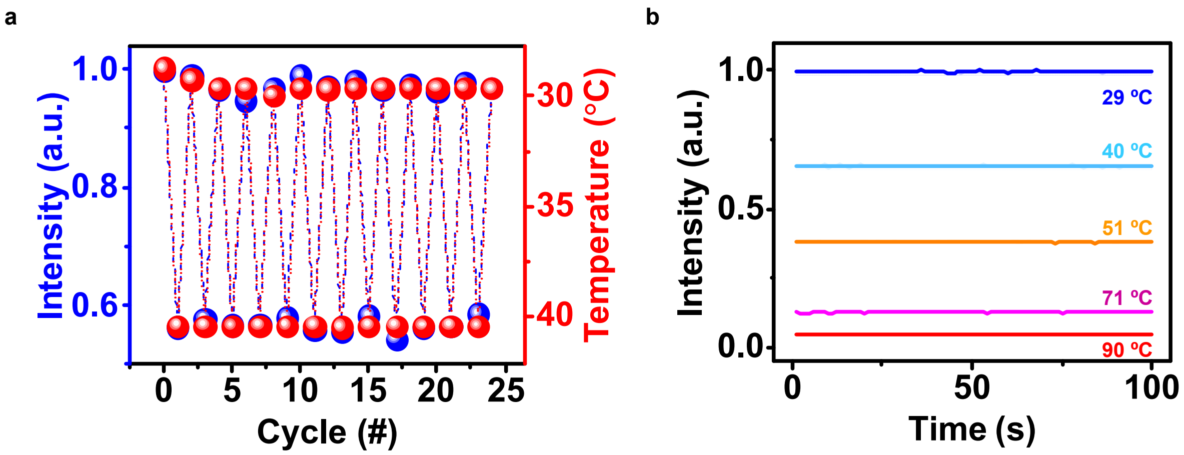

Figure S9. (a) Measured PL intensity and corresponding temperature values under cyclic temperature changes between 28 ºC and 41 ºC. (b) The stability of the optoelectronic upconversion device at 29 ºC, 40 ºC, 51 ºC, 71 ºC, and 90 ºC, after the heating plate stabilizes.

Figure S10

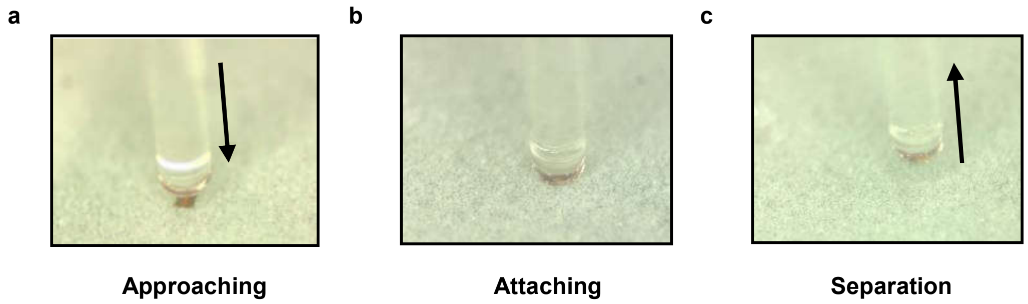

Figure S10. Transfer printing a free-standing microscale upconversion device from a thermal release tape to the optical fiber tip.

Figure S11

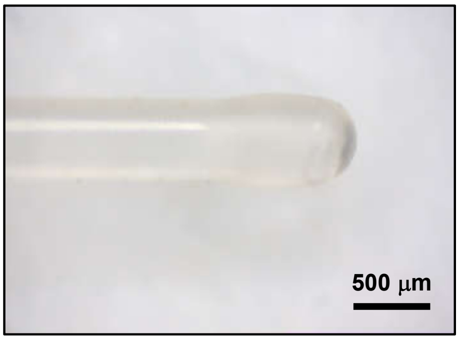

Figure S11. Cross-sectional view of the microscope image for the optoelectronic upconversion device integrated on the fiber optics, in which the fiber tip is encapsulated with a bilayer of parylene (~10 µm) and PDMS (~20 µm) .

Figure S12

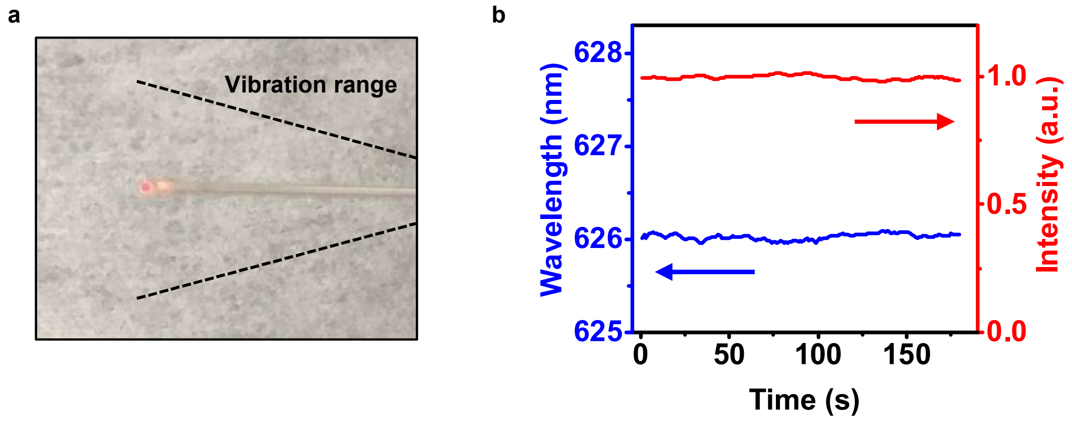

Figure S12. Stability of PL signals recorded from the fiber sensor under vibration conditions, in which the fiber bending angle range is about +/−30º.

Figure S13

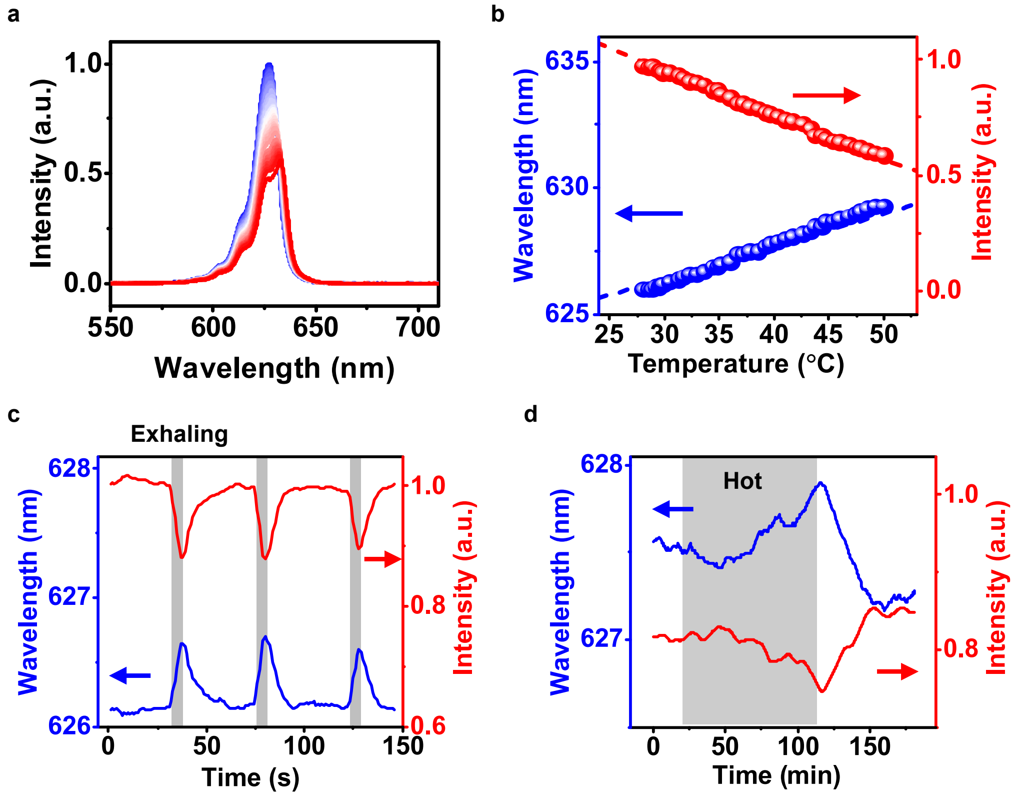

Figure S13. (a) PL emission recorded from the fiber sensor at different temperatures (25–50 ºC) in PBS. (b) Calculated (dash line) and measured (dots) emission peak wavelength and PL intensity as a function of temperature. (c) Recorded PL peak wavelength and intensity from the fiber sensor, in response to human respiration. Data are used to plot Figure 4e. (d) Recorded PL peak wavelength and intensity from the fiber sensor, obtained in the mouse brain. Data are used to plot Figure 4h.

Figure S14

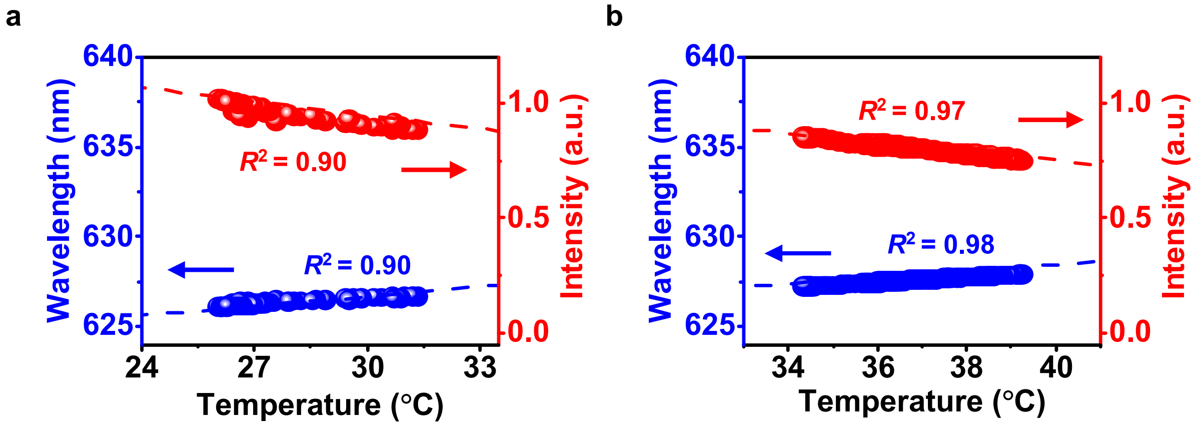

Figure S14. Calculated (dash line) and measured (dots) peak wavelength and PL intensity of the upconverted emission as a function of temperature, corresponding to results in (a) human respiration in Figure 4e and (b) the mouse brain in Figure 4h.

Movie S1

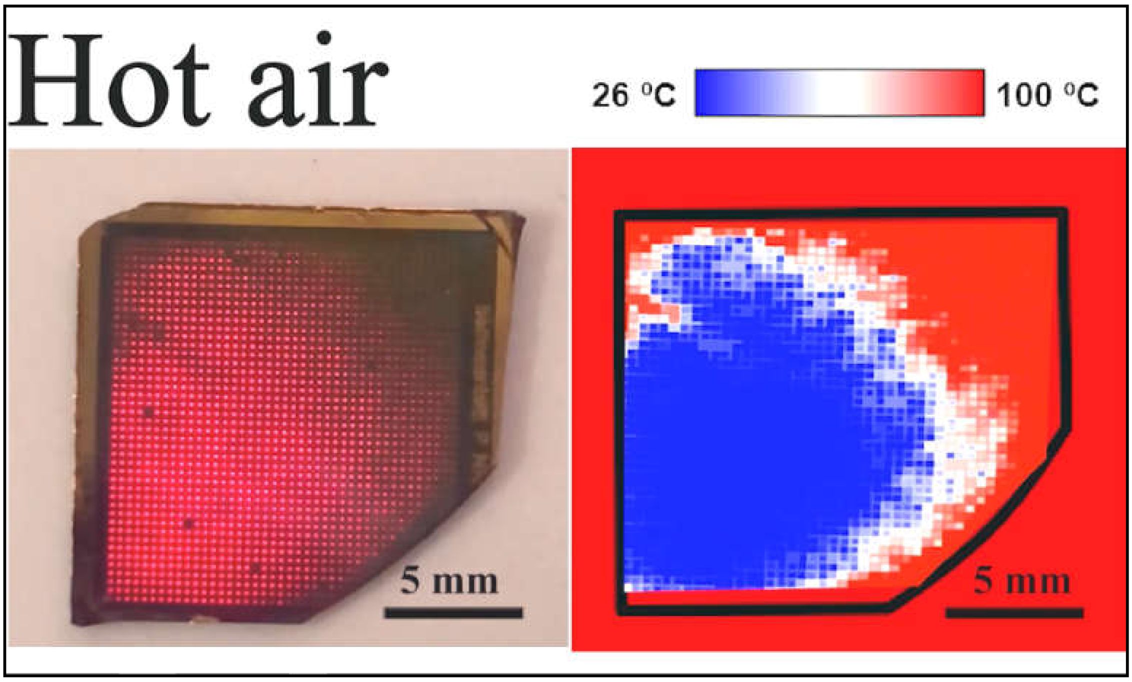

Movie S1. Spatially resolved dynamic PL variations in response to temperature changes based on an optoelectronic upconversion device array.

Movie S2

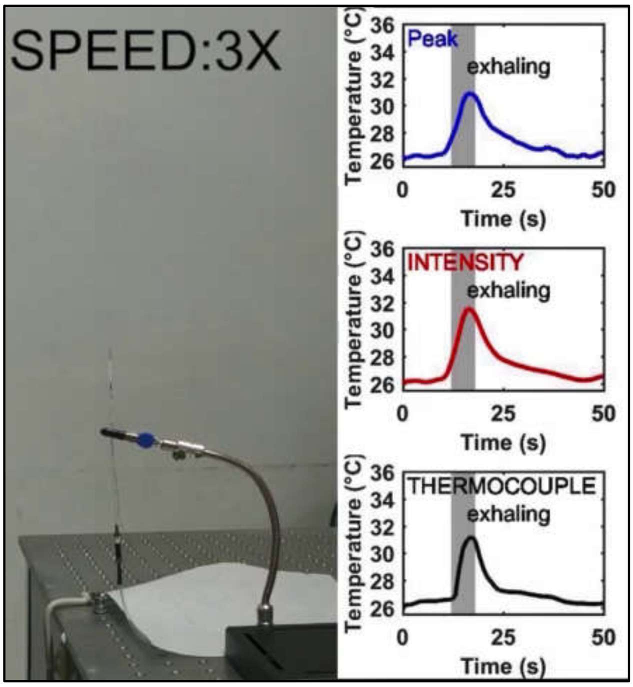

Movie S2. Dynamic temperature signals during exhaling activities obtained by the fiber sensor based on the emission peak wavelength shifts and PL intensity changes, compared with results simultaneously recorded by the thermocouple.
